# The Relationships Between Hyperprolactinemia, Metabolic Disturbance, and Sexual Dysfunction in Patients With Schizophrenia Under Olanzapine Treatment

**DOI:** 10.3389/fphar.2021.718800

**Published:** 2021-08-05

**Authors:** Tzu-Hua Wu, Chieh-Hsin Lin, Kah Kheng Goh, Cynthia Yi-An Chen, Chun-Hsin Chen, Hsien-Yuan Lane, Mong-Liang Lu

**Affiliations:** ^1^ Department of Clinical Pharmacy, School of Pharmacy, College of Pharmacy, Taipei Medical University, Taipei, Taiwan; ^2^ Psychiatric Research Center, Wan Fang Hospital, Taipei Medical University, Taipei, Taiwan; ^3^ Department of Psychiatry, Kaohsiung Chang Gung Memorial Hospital, Chang Gung University College of Medicine, Kaohsiung, Taiwan; ^4^ Graduate Institute of Biomedical Sciences, China Medical University, Taichung, Taiwan; ^5^ School of Medicine, Chang Gung University, Taoyuan, Taiwan; ^6^ Department of Psychiatry, Wan Fang Hospital, Taipei Medical University, Taipei, Taiwan; ^7^ Department of Psychiatry, School of Medicine, College of Medicine, Taipei Medical University, Taipei, Taiwan; ^8^ Department of Psychiatry and Brain Disease Research Center, China Medical University Hospital, Taichung, Taiwan

**Keywords:** olanzapine, metabolic syndrome, prolactin, sexual dysfunction, schizophrenia

## Abstract

The aim of the study was to assess the relationship between prolactin levels and sexual dysfunction in patients with schizophrenia who use olanzapine medication. The potential risk factors of hyperprolactinemia and sexual dysfunction were also investigated. Patients with schizophrenia undergoing olanzapine monotherapy were invited to participate in this cross-sectional study. The Arizona Sexual Experiences Scale (ASEX) and the Positive and Negative Syndrome Scale were used to evaluate subjective sexual dysfunction and psychopathology, respectively. Levels of prolactin and metabolic parameters were also measured. In total, 279 participants with schizophrenia were recruited. The overall incidences of hyperprolactinemia, sexual dysfunction, and metabolic syndrome were 51.6, 53.8, and 43.7%, respectively. Higher ASEX scores, higher insulin levels, female sex, and younger age were associated with hyperprolactinemia. Prolactin level was significantly correlated with ASEX score. Elevated prolactin levels, concomitant antidepressant, increased insulin resistance, longer illness duration, and female sex were associated with sexual dysfunction. Female participants recorded higher levels of sexual dysfunction than their male counterparts did, whereas male participants had comparatively lower prolactin levels and lower rates of spousal partnership. Hyperprolactinemia, metabolic syndrome, and sexual dysfunction are prevalent in patients with schizophrenia treated with olanzapine. Clinicians should maintain awareness of these problems and monitor them regularly with their patients.

## Introduction

Schizophrenia is a severe and chronic mental disorder that causes marked functional impairment. Antipsychotic medication is the first-line treatment for patients with schizophrenia. However, antipsychotic drugs may produce unwanted side effects due to their pharmacodynamic characteristics. Antipsychotics often elevate prolactin levels by blocking dopamine D_2_ receptors in the tuberoinfundibular pathway of the brain (Grigg et al., 2017). As a potent antagonist of dopamine D_2_ and serotonin 5-HT_2_ receptors, olanzapine has fewer extrapyramidal side effects and better clinical efficacy compared with first-generation antipsychotics (Callaghan et al., 1999). But several studies reported that olanzapine was associated with elevated prolactin levels to some degree (Barata et al., 2019; Huhn et al., 2019). It is proposed that because 5-HT_2_ receptor stimulation can induce prolactin release, the 5-HT_2_ receptor antagonism of olanzapine may partially counteract its prolactin-elevating tendency through its D2 receptor antagonism (Cowen et al., 1990).

Sexual dysfunction is characterized by a significant disturbance in a person’s ability to respond sexually or experience sexual pleasure. Finn et al. (1990) reported that patients with schizophrenia rated antipsychotic-induced sexual dysfunction as more “bothersome” than most psychiatric symptoms of their illness are. The prevalence of sexual dysfunction among people with schizophrenia is higher than that among the rest of the population. Sexual dysfunction has been reported in 30–82% of patients with psychiatric disorders who use antipsychotics, due to hyperprolactinemia as well as α1 and dopamine D_2_ receptor blockades (De Boer et al., 2015; Dumontaud et al., 2020). A meta-analysis reported that olanzapine users had a higher rate of sexual dysfunction (approximately 40%) as compared with the prolactin-sparing antipsychotics quetiapine, ziprasidone, and aripiprazole (16–27%) (Serretti and Chiesa, 2011). The causal relationship between hyperprolactinemia and sexual dysfunction remains contested. Some studies have suggested a relationship between the disorders (Ahl et al., 2004; Rubio-Abadal et al., 2016), but other studies have discovered no such relationship (Johnsen et al., 2011; Kikuchi et al., 2012). Other factors that have been suggested to be related to sexual dysfunction in patients with schizophrenia include sex, age, disease psychopathology, effects of other medications, substance use, and medical comorbidities (Martin et al., 2018; Dehelean et al., 2020; Dumontaud et al., 2020). Antipsychotic-induced sexual dysfunction can significantly distress patients and subsequently reduce drug compliance (Souaiby et al., 2019); accordingly, evaluating sexual dysfunction and managing it properly are essential.

Metabolic disturbance is a common adverse reaction in patients with schizophrenia under olanzapine treatment (Hirsch et al., 2017; Lu et al., 2018). Several studies reported that metabolic dysfunction is associated with sexual dysfunction in general population (Schulster et al., 2017; Di Francesco et al., 2019). But the relationships between metabolic syndrome and sexual dysfunction in patients with schizophrenia have been poorly explored (Dumontaud et al., 2020).

Our study was aimed to examine the relationships between hyperprolactinemia, metabolic disturbance, and sexual dysfunction among study participants with schizophrenia who received olanzapine medication. Potential links to hyperprolactinemia and sexual dysfunction were also analyzed.

## Materials and Methods

### Participants

This cross-sectional study was approved by our facility’s institutional review board. After providing a detailed description of the study, we obtained written informed consent from the participants. Patients aged 20–65 years and with schizophrenia as diagnosed according to the *Diagnostic and Statistical Manual of Mental Disorders*, fifth edition, were included in the study. They were required to have undergone olanzapine monotherapy at the same dose for the last 6 months.

### Assessment

All study participants participated in clinical interviews, underwent anthropometrical parameter assessments, and provided fasting blood samples. A trained research nurse interviewed patients to collect demographic and psychiatric information. Body mass index was calculated as weight in kilograms divided by the square of the height in meters (kg/m^2^).

The Arizona Sexual Experiences Scale (ASEX) was used to assess sexual dysfunction (Mcgahuey et al., 2000). ASEX is a five-item rating scale that assesses sex drive, arousal, vaginal lubrication/penile erection, ability to reach orgasm, and satisfaction from orgasm. It measures sexual dysfunction on a 6-point Likert scale ranging from hyperfunction (1) to hypofunction (6). A participant with a total ASEX score of ≥19, any one item with a score of ≥5, or any 3 items with a score of ≥4 was deemed to be experiencing sexual dysfunction. We also assessed the participants for psychopathology severity by using the Positive and Negative Syndrome Scale (Kay et al., 1987).

Blood samples were obtained in the morning after overnight fasting. Plasma was stored at −80°C prior to testing. Levels of prolactin were measured using electrochemiluminescence immunoassays. Hyperprolactinemia was considered when the prolactin level exceeded 20 ng/ml in women and 15 ng/ml in men, in accordance with laboratory procedures. Enzymatic colorimetric assays were used to measure fasting serum levels of glucose, high-density lipoprotein cholesterol (HDL-C), and triglycerides. Serum insulin levels were measured using an electrochemiluminescence immunoassay kit. Insulin resistance was calculated using the homeostasis model assessment for insulin resistance (HOMA-IR) as follows (fasting glucose [mmol/L] × fasting insulin [mU/L]/22.5) (Matthews et al., 1985).

In this study, we used the modified Adult Treatment Panel III criteria for Asians to evaluate participants for metabolic syndrome (Tan et al., 2004). A diagnosis of metabolic syndrome required 3 of the following five criteria: (1) abdominal obesity (waist circumference of ≥90 cm in men and ≥80 cm in women), (2) fasting hypertriglyceridemia (≥150 mg/dl); (3) low fasting HDL-C levels (<40 mg/dl in men and <50 mg/dl in women), (4) high blood pressure (≥130/≥85 mm Hg) or current treatment with antihypertensive medication, and (5) high fasting levels of plasma glucose (≥100 mg/dl) or current treatment with antidiabetic medication.

### Statistical Analysis

The variables were compared using the Student’s *t* test for continuous variables and Fisher’s exact test for categorical variables. Nonparametric tests (Mann–Whitney U test) were performed for variables without normal distribution. We used the Pearson correlation method to analyze the correlations between variables and ASEX scores as well as prolactin levels. A multivariate linear regression model with selection of clinically relevant variables related to hyperprolactinemia and sexual dysfunction was utilized. All continuous variables that were entered in the regression were logarithmically transformed to normalize the data, whereas categorical variables were recoded into sets of distinct binary variables. The significance level used was *p* < 0.05. Analyses were performed using SPSS 19.

## Results

In total, 279 participants with schizophrenia, comprising 147 women and 132 men, were recruited for our study. The demographic, clinical, and laboratory data of the study participants with and without hyperprolactinemia are summarized in Table 1. The overall prevalence of hyperprolactinemia was 51.6%. Compared with participants not diagnosed as having hyperprolactinemia, those with hyperprolactinemia were younger, were less likely to have a spouse or partner, and recorded higher ASEX scores, prevalence of sexual dysfunction, insulin levels, and HOMA-IR indexes.

**TABLE 1:** Demographic, clinical and laboratory characteristics of study subjects by hyperprolactinemia.

	Hyperprolactinemia (+) (n = 144)	Hyperprolactinemia (-) (n = 135)	*p* value
**Demographic parameters**			
Sex (male/female)	67/77	65/70	0.811
Age (years)	41.5 ± 12.8	44.3 ± 9.9	**0.040**
Duration of illness (years)	14.3 ± 10.3	15.9 ± 9.0	0.168
Tobacco use	30.6%	32.6%	0.797
Marital status			
Married/With partner	23	38	**0.020**
Single/Divorced/Widowed	121	97	
BMI	26.0 ± 5.4	25.3 ± 4.1	0.212
Waist circumference (cm)	88.3 ± 13.5	87.5 ± 9.9	0.600
SBP (mmHg)	120.1 ± 15.8	121.4 ± 13.3	0.452
DBP (mmHg)	76.1 ± 11.2	76.6 ± 9.9	0.686
**Concomitant medication**			
Benzodiazepine	42.4%	50.4%	0.188
Anticholinergics	25.7%	31.1%	0.353
Antidepressant	8.3%	11.9%	0.426
**Clinical parameters**			
Olanzapine dose	13.8 ± 5.5	14.6 ± 5.6	0.215
PANSS positive score	15.6 ± 5.3	15.1 ± 5.4	0.432
PANSS negative score	15.7 ± 5.5	15.5 ± 5.3	0.762
PANSS general score	27.5 ± 7.7	26.8 ± 7.8	0.496
PANSS total score	58.8 ± 15.5	57.5 ± 15.3	0.468
ASEX score	22.0 ± 5.1	14.3 ± 4.4	**<0.001**
Sexual dysfunction	79.9%	25.9%	**<0.001**
**Laboratory parameters**			
Glucose (mg/dl)	92.8 ± 17.7	96.9 ± 39.9	0.269
Insulin (μU/mL)	15.8 ± 17.5	9.7 ± 7.8	**<0.001**
HOMA-IR	4.1 ± 6.1	2.4 ± 2.4	**0.003**
Triglycerides (mg/dl)	157.9 ± 105.3	153.1 ± 89.8	0.681
HDL-C (mg/dl)	49.5 ± 16.5	50.0 ± 17.4	0.816
Metabolic syndrome	42.4%	45.2%	0.717

Values in bold indicate statistically significant results (*p* < 0.05). ASEX, arizona sexual experiences scale; BMI, body mass index; DBP, diastolic blood pressure; HDL-C, high-density lipoprotein cholesterol; HOMA-IR, homeostasis model assessment for insulin resistance; PANSS, positive and negative syndrome Scale; SBP, systolic blood pressure.

A multivariate liner regression model was applied to identify the variables associated with hyperprolactinemia. For all participants with schizophrenia, ASEX score (B = 0.053, *p* < 0.001), age (B = −0.006, *p* = 0.004), female sex (B = 0.119, *p* = 0.015), and insulin level (B = 0.014, *p* = 0.044) were associated with hyperprolactinemia (Table 2).

**TABLE 2:** Multiple linear regression analysis with hyperprolactinemia as dependent variable.

	Hyperprolactinemia		
	B	95% CI	*p* value
ASEX score	0.053	0.046 to 0.061	**< 0.001**
Female sex	0.119	0.024 to 0.214	**0.015**
Age (years)	−0.006	−0.010 to −0.002	**0.004**
Duration of illness (years)	0.000	−0.005 to 0.005	0.861
Married/With partner	−0.004	−0.119 to 0.112	0.952
Concomitant antidepressant	−0.035	−0.189 to 0.118	0.649
Olanzapine dose	−0.004	−0.012 to 0.004	0.357
PANSS total score	−0.001	−0.004 to 0.002	0.654
Glucose (mg/dl)	−0.001	−0.003 to 0.001	0.334
Insulin (μU/mL)	0.014	0.000 to 0.029	**0.044**
HOMA-IR	−0.028	−0.070 to 0.015	0.197
Metabolic syndrome	−0.071	−0.171 to 0.029	0.164

Values in bold indicate statistically significant results (*p* < 0.05). ASEX, arizona sexual experiences scale; HOMA-IR, homeostasis model assessment for insulin resistance; PANSS, positive and negative syndrome scale.

**FIGURE 1:**
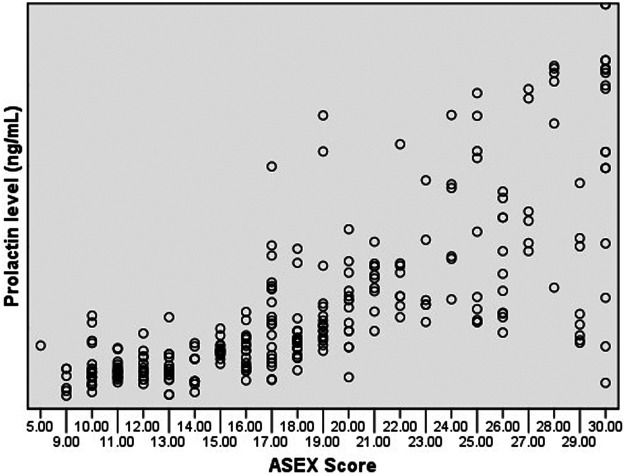
The scatter plot of ASEX score and prolactin level.

Prolactin level was significantly correlated with ASEX score (*r* = 0.689, *p* < 0.001) (Figure 1). And prolactin level was also positively correlated with each ASEX item score (all *p* < 0.001). Other demographic parameters, clinical parameters, or metabolic parameters were not correlated with prolactin level.

The demographic, clinical, and laboratory characteristics of the study participants with and without sexual dysfunction are summarized in Table 3. The overall prevalence of sexual dysfunction was 53.8%. Compared with participants without sexual dysfunction, participants with sexual dysfunction were more likely to be female and advanced in age and more likely to have had concomitant antidepressant, longer durations of illness, and higher prolactin levels. Regarding metabolic parameters, participants with sexual dysfunction had higher glucose levels, insulin levels, and insulin resistance compared with those not experiencing sexual dysfunction. No differences in psychopathology or olanzapine dose between those two groups were observed.

**TABLE 3:** Demographic, clinical and laboratory characteristics of study subjects by sexual dysfunction.

	Sexual dysfunction (+) (n = 150)	Sexual dysfunction (-) (n = 129)	*p* value
**Demographic parameters**			
Sex (male/female)	54/96	78/51	**<0.001**
Age (years)	44.2 ± 12.7	41.3 ± 9.7	**0.032**
Duration of illness (years)	16.6 ± 10.8	13.3 ± 7.9	**0.004**
Tobacco use	27.3%	36.4%	0.121
Marital status			
Married/With partner	27	34	0.110
Single/Divorced/Widowed	123	95	
BMI	25.7 ± 5.2	25.6 ± 4.3	0.756
Waist circumference (cm)	87.9 ± 12.3	87.9 ± 11.4	0.952
SBP (mmHg)	121.1 ± 14.9	120.4 ± 14.4	0.676
DBP (mmHg)	76.7 ± 10.5	76.0 ± 10.6	0.532
**Concomitant medication**			
Benzodiazepine	47.3%	45.0%	0.719
Anticholinergics	30.7%	25.6%	0.355
Antidepressant	15.3%	3.9%	**0.001**
**Clinical parameters**			
Olanzapine dose	14.0 ± 5.5	14.3 ± 5.5	0.669
PANSS positive score	15.5 ± 5.0	15.3 ± 5.8	0.709
PANSS negative score	16.0 ± 5.6	15.1 ± 5.2	0.167
PANSS general score	27.9 ± 8.1	26.2 ± 7.2	0.067
PANSS total score	59.5 ± 15.7	56.7 ± 14.9	0.124
ASEX score	22.6 ± 4.5	13.2 ± 3.4	**<0.001**
**Laboratory parameters**			
Prolactin level (ng/ml)	35.6 ± 25.1	13.4 ± 9.9	**<0.001**
Hyperprolactinemia	76.7%	22.5%	**<0.001**
Glucose (mg/dl)	98.2 ± 28.4	90.9 ± 32.5	**0.049**
Insulin (μU/mL)	15.1 ± 17.4	10.2 ± 8.0	**0.002**
HOMA-IR	4.0 ± 6.1	2.4 ± 2.1	**0.003**
Triglycerides (mg/dl)	153.1 ± 93.3	158.4 ± 103.3	0.659
HDL-C (mg/dl)	51.2 ± 16.2	48.1 ± 17.6	0.125
Metabolic syndrome	46.7%	40.3%	0.333

Values in bold indicate statistically significant results (*p* < 0.05). ASEX, arizona sexual experiences scale; BMI, body mass index; DBP, diastolic blood pressure; HDL-C, high-density lipoprotein cholesterol; HOMA-IR, homeostasis model assessment for insulin resistance; PANSS, positive and negative syndrome scale; SBP, systolic blood pressure.

A multivariate regression model was used to identify the factors associated with sexual dysfunction. For all participants with schizophrenia, prolactin level (B = 0.012, *p* < 0.001), concomitant antidepressant (B = 0.460, *p* < 0.001), HOMA-IR (B = 0.018, *p* = 0.001), duration of illness (B = 0.007, *p* = 0.008), and female sex (B = 0.0.127, *p* = 0.013) were associated with sexual dysfunction (Table 4).

**TABLE 4:** Multiple linear regression analysis with sexual dysfunction as dependent variable.

	Sexual dysfunction		
	B	95% CI	*p* value
Prolactin level (ng/ml)	0.012	0.009 to 0.014	**< 0.001**
Female sex	0.127	0.027 to 0.227	**0.013**
Age (years)	0.001	−0.004 to 0.005	0.773
Duration of illness (years)	0.007	0.002 to 0.012	**0.008**
Married/With partner	−0.011	−0.126 to 0.104	0.850
Concomitant antidepressant	0.460	0.296 to 0.623	**< 0.001**
Olanzapine dose	−0.005	−0.013 to 0.003	0.265
PANSS total score	0.000	−0.003 to 0.004	0.785
Glucose (mg/dl)	0.001	−0.001 to 0.003	0.360
Insulin (μU/mL)	−0.004	−0.019 to 0.011	0.602
HOMA-IR	0.018	0.007 to 0.028	**0.001**
Metabolic syndrome	0.010	−0.096 to 0.116	0.848

Values in bold indicate statistically significant results (*p* < 0.05). HOMA-IR, homeostasis model assessment for insulin resistance; PANSS, positive and negative syndrome scale.

With total and individual ASEX item scores as dependent variables, multivariate regression analyses were performed to find the associated factors. Prolactin level was positively associated with total ASEX score and all five item scores (Table 5). Female sex was positively associated with total ASEX score as well as scores of ASEX item 2 (ease of arousal), item 3 (ability to achieve erection/lubrication), and item 4 (ease of reaching orgasm). Concomitant antidepressant was positively associated with total ASEX score as well as scores of item 3 and item 4. Married participants or participants with sex partner were negatively associated with scores of ASEX item 3 and item 4.

**TABLE 5:** Multiple linear regression analysis with individual ASEX item scores as dependent variables.

	Total ASEX score		Item 1 sex drive		Item 2 ease of arousal		Item 3 ability to achieve erection/Lubrication		Item 4 ease of reaching orgasm		Item 5 orgasm satisfaction	
	B		B	*p* value	B	*p* value	B	*p* value	B	*p* value	B	*p* value
Prolactin level (ng/ml)	0.212	**<0.001**	0.043	**< 0.001**	0.040	**<0.001**	0.042	**<0.001**	0.043	**<0.001**	0.044	**<0.001**
Female sex	1.37	**0.010**	0.165	0.213	0.322	**0.015**	0.415	**0.002**	0.331	**0.008**	0.143	0.258
Age (years)	0.000	0.983	0.003	0.573	−0.003	0.647	0.005	0.379	−0.003	0.607	−0.004	0.431
Duration of illness (years)	0.011	0.382	0.002	0.810	0.006	0.422	0.007	0.296	−0.001	0.916	−0.003	0.705
Married/With partner	−1.181	0.069	-0.026	0.872	−0.244	0.126	−0.346	**0.033**	−0.382	**0.011**	−0.230	0.134
Concomitant antidepressant	1.991	**0.022**	0.312	0.150	0.353	0.100	0.578	**0.008**	0.531	**0.009**	0.255	0.216
Olanzapine dose	−0.061	0.188	−0.009	0.454	−0.006	0.596	−0.019	0.098	−0.021	0.051	−0.005	0.623
PANSS total score	0.002	0.922	0.000	0.996	0.002	0.659	−0.004	0.391	0.001	0.774	0.003	0.426
Glucose (mg/dl)	−0.002	0.824	−0.001	0.691	0.004	0.168	−0.002	0.516	−0.001	0.826	−0.003	0.296
Insulin (μU/mL)	−0.057	0.482	0.009	0.654	−0.003	0.866	−0.027	0.186	−0.022	0.241	−0.011	0.249
HOMA-IR	0.371	0.183	0.051	0.289	0.081	0.169	0.102	0.065	0.076	0.178	0.061	0.257
Metabolic syndrome	0.412	0.465	−0.035	0.802	−0.036	0.796	0.012	0.935	0.216	0.121	0.154	0.249

Values in bold indicate statistically significant results (*p* < 0.05). ASEX, arizona sexual experiences scale; HOMA-IR, homeostasis model assessment for insulin resistance; PANSS, positive and negative syndrome scale.

Among all parameters, only prolactin level was correlated with the ASEX score (r = 0.689, *p* < 0.001).

The demographic, clinical, and laboratory information of the participants by sex are summarized in Supplementary Table S1. Compared with female participants, male participants were more likely to be young and smoke, as well as less likely to have a spouse or partner. Male participants also recorded lower ASEX scores, lower prevalence of sexual dysfunction, lower prolactin levels, lower glucose levels, lower HDL-C levels, larger waist circumferences, and higher blood pressure values. No differences between sexes in the severity of psychopathology or olanzapine dose were observed.

## Discussion

In our study, approximately half of the olanzapine-treated participants with schizophrenia had hyperprolactinemia and sexual dysfunction. Hyperprolactinemia was associated with sexual dysfunction, female sex, higher insulin level, and younger age. Sexual dysfunction was significantly related to higher prolactin levels in olanzapine-treated patients with schizophrenia, with significant differences between the sexes. Concomitant antidepressant, longer durations of illness, and greater insulin resistance were also associated with sexual dysfunction.

Given that prolactin is under negative control by dopamine and positive control by serotonin, olanzapine has been reported to induce moderately elevated prolactin levels in patients with schizophrenia (Peuskens et al., 2014). In our study, the mean prolactin level was 25.3 ± 22.5 ng/ml in all subjects and 51.6% of participants had hyperprolactinemia. Consistent with our result, other study data have revealed prevalence rates of olanzapine-induced hyperprolactinemia ranging between 6 and 60% (Peuskens et al., 2014). However, our results demonstrated that no dose-dependent effect of olanzapine on prolactin levels in patients with schizophrenia exists. Some studies have supported the dose-dependent effect of olanzapine on plasma prolactin levels (Kinon et al., 2008; Citrome et al., 2009; Suzuki et al., 2011), but others have not (Karagianis and Baksh, 2003; Takeuchi et al., 2014).

The prevalence of sexual dysfunction reported by participants in our study was 53.8%. In Taiwan, the prevalence of sexual dysfunction among men and women aged 40–80 years was observed to be 32 and 51%, respectively (Nicolosi et al., 2005). The rates of sexual dysfunction reported by participants with schizophrenia are higher than those of the general population (De Boer et al., 2015; Zhao et al., 2020). Studies using structured interviews or self-report questionnaires have noted the prevalence of antipsychotic-associated sexual side effects in 30–60% of participants (De Boer et al., 2015). Postsynaptic dopamine blockade, prolactin elevation, and α1-receptor antagonism might be the underlying mechanisms in the pathogenesis of antipsychotic-induced sexual dysfunction (De Boer et al., 2015). Our result supports the findings of studies that have indicated that, among second-generation antipsychotics, olanzapine is moderately associated with the increased prevalence of sexual dysfunction (Dumontaud et al., 2020).

Our study supports that the association between higher prolactin levels and sexual dysfunction in patients with schizophrenia (De Hert et al., 2014). Hyperprolactinemia has profound effects on reproductive health and sexual function, including hypogonadism, decreased libido in both sexes, amenorrhea and infertility in women and low sperm count and reduced muscle mass in men. It is proposed that prolactin elevation might reduce sex hormone release *via* an alteration of the hypothalamic–pituitary–gonadal axis, and consequently lead to sexual dysfunction (Smith et al., 2002).

We found concomitant antidepressant was associated sexual dysfunction. Furthermore, concomitant antidepressant was associated with inability to achieve erection/lubrication and difficulty of reaching orgasm. One possibility is that antidepressant itself induced sexual dysfunction (Clayton et al., 2016). Another possibility is that antidepressant prescription might imply subjects had depressive symptoms. Depressive symptomatology in patients with schizophrenia may contribute to sexual dysfunction (Dumontaud et al., 2020).

We ascertained that patients with longer duration of illness are at greater risk of sexual dysfunction. The relationship between longer illness duration and sexual dysfunction is likely mediated by extended exposure to antipsychotic treatments and the characteristics of the chronic illness itself (Lee et al., 2015). Some studies, however, have reported that longer antipsychotic treatment duration is not associated with sexual dysfunction (Souaiby et al., 2019). Differences between those in the literature may be attributable to the differences in the current and past antipsychotic medications as well as the characteristics of study subjects. But duration of illness was not associated with total ASEX score or individual ASEX item scores in our study. Further studies are warranted to investigate the relationship between sexual dysfunction and duration of illness in patients with schizophrenia.

A difference between the sexes in the prevalence of sexual dysfunction among patients with schizophrenia is inconsistent in previous studies; some authors have reported higher rates of sexual dysfunction in female patients (Bianco et al., 2019; Huang et al., 2019), others have reported the opposite (Cutler, 2003), whereas some have discovered similar rates for both sexes (Fujii et al., 2010). Our study found that female sex was associated with sexual dysfunction in olanzapine-treated patients with schizophrenia. And female sex was positively associated with total ASEX score as well as difficulty of arousal, inability to achieve erection/lubrication, and difficulty of reaching orgasm. Epidemiologic study reported that the prevalence of sexual dysfunction was higher in women (40–45%) than men (20–30%) (Lewis et al., 2004). In the present study, female patients exhibited significantly higher serum levels of prolactin and metabolic parameters than male patients. Those differences between male and female patients may contribute to gender-related differences in sexual dysfunction.

We found that subjects with hyperprolactinemia had higher insulin levels and HOMA-IR indices than those without hyperprolactinemia. Prolactin is not only a lactogenic hormone; it possesses more than 300 physiological effects, including an influence on metabolism (Andersen and Glintborg, 2018). Several large cohort studies among the general population have concluded that low prolactin levels are associated with metabolic disease and represent a risk factor for type 2 diabetes (Macotela et al., 2020). However, other population studies have reported contradictory results (Balbach et al., 2013; Daimon et al., 2017). In patients with schizophrenia spectrum psychosis, a relationship between hyperprolactinemia and insulin resistance was discovered (Petruzzelli et al., 2018). The effects of prolactin on metabolic homeostasis may be different when prolactin levels are within and outside the physiological range (Daimon et al., 2017; Andersen and Glintborg, 2018). Furthermore, Gragnoli et al. hypothesized that the dopamine–prolactin pathway potentially contributes to the comorbidity of schizophrenia and type 2 diabetes (Gragnoli et al., 2016).

Consistent with studies in general population (Schulster et al., 2017; Di Francesco et al., 2019), we found that subjects with sexual dysfunction had higher HOMA-IR indices than those without sexual dysfunction. However, the underlying mechanisms of how insulin resistance results in sexual dysfunction remain unclear. Insulin resistance plays an important role in the pathogenesis of inflammation and endothelial dysfunction which subsequently progress to cardiovascular disease (Muniyappa and Sowers, 2013). In men, insulin resistance impaired vascular nitric oxide production and insulin-induced vasodilation, both of which are likely to cause sexual dysfunction (Schulster et al., 2017; Maiorino et al., 2018). In women, insulin resistance decreased nitric oxide production and vascular vaginal relaxation, both of which might cause sexual dysfunction (Rahmanian et al., 2019). In addition, insulin had an inhibitory effect on the production of sex hormone-binding globulin by the liver (Wallace et al., 2013). Those might explain that insulin resistance plays an important role in the pathogenesis of sexual dysfunction. But HOMA-IR indices were not associated with total ASEX scores or individual ASEX item scores in our study. Further studies are warranted to investigate the relationship between sexual dysfunction and insulin resistance in patients with schizophrenia.

In this study, participants who were married or with sex partner were negatively associated with inability to achieve erection/lubrication and difficulty of reaching orgasm. Consistent with our result, other studies have reported that marital status was positively associated with sexual arousal and sexual satisfaction (Lee et al., 2015; Fanta et al., 2018). The present findings may be attributed to the fact that married subjects are more likely to have the opportunity to engage in sexual relationship and activity. However, marital status was not associated with sexual dysfunction in this study. Further studies with more comprehensive assessment of sexual function to investigate the role of marital status are warranted.

Some studies have reported a relationship between sexual dysfunction and the severity of psychotic symptoms (Kheng Yee et al., 2014; Simiyon et al., 2016), whereas others did not ascertain such an association (Martin et al., 2018). In this study, we did not determine sexual dysfunction to be associated with the severity of psychopathology. Although we did not exclude patients with severe psychopathology, our naturalistic study design may have unintentionally entailed an inclusion bias favoring participants who were stable psychopathologically.

Consistent with other studies (Yasui-Furukori et al., 2010), male participants in our study recorded comparatively lower prolactin levels than female participants did. This might be partly due to higher base levels of prolactin and higher vulnerability to hyperprolactinemia in response to antipsychotic drugs in women (Kinon et al., 2003). The lower prolactin levels in male participants might partially explain their lower ASEX scores and rates of sexual dysfunction. In concordance the research of Häfner (2002), our study also recorded a higher rate of marriage and partnership among female participants compared with the male participants.

Several limitations were present in our study. First, because of the cross-sectional design of this study, potential selection and confounding biases should be assumed and a causal relationship between hyperprolactinemia and sexual dysfunction cannot be concluded. Second, the exact days in the female participants’ menstrual cycles when the blood tests were performed were not recorded in our study. Third, the absence of healthy controls or drug-naïve patients with schizophrenia in our study rendered the differentiation of the effects on disease and medication impossible. Fourth, patients with schizophrenia in this study were clinically stable; hence, the results cannot be generalized to patients in other illness stages.

## Conclusion

In conclusion, approximately half of the olanzapine-treated patients with schizophrenia in our study had hyperprolactinemia, metabolic syndrome, and sexual dysfunction. Higher prolactin levels, concomitant antidepressant, greater insulin resistance indices, longer duration of illness, and female sex were associated with sexual dysfunction. Prolactin levels, metabolic parameters, and sexual function in olanzapine-treated patients with schizophrenia should be monitored regularly.

## Data Availability

The raw data supporting the conclusions of this article will be made available by the authors, without undue reservation.

## References

[B1] Ahl J. Kinon B. J. Liu-Seifert H. (2004). Sexual Dysfunction Associated with Neuroleptic-Induced Hyperprolactinemia Improves with Reduction in Prolactin Levels. Ann. N. Y Acad. Sci. 1032, 289–290. 10.1196/annals.1314.041 15677431

[B2] Andersen M. Glintborg D. (2018). Metabolic Syndrome in Hyperprolactinemia. Front. Horm. Res. 49, 29–47. 10.1159/000486000 29894997

[B3] Balbach L. Wallaschofski H. Volzke H. Nauck M. Dorr M. Haring R. (2013). Serum Prolactin Concentrations as Risk Factor of Metabolic Syndrome or Type 2 Diabetes? BMC Endocr. Disord. 13, 12. 10.1186/1472-6823-13-12 23517652 PMC3614874

[B4] Barata P. C. Santos M. J. Melo J. C. Maia T. (2019). Olanzapine-Induced Hyperprolactinemia: Two Case Reports. Front. Pharmacol. 10, 846. 10.3389/fphar.2019.00846 31417404 PMC6680598

[B5] Bianco C. L. Pratt S. I. Ferron J. C. (2019). Deficits in Sexual Interest Among Adults with Schizophrenia: Another Look at an Old Problem. Ps 70, 1000–1005. 10.1176/appi.ps.201800403 31401908

[B6] Callaghan J. T. Bergstrom R. F. Ptak L. R. Beasley C. M. (1999). Olanzapine. Clin. Pharmacokinet. 37, 177–193. 10.2165/00003088-199937030-00001 10511917

[B7] Citrome L. Stauffer V. L. Chen L. Kinon B. J. Kurtz D. L. Jacobson J. G. (2009). Olanzapine Plasma Concentrations after Treatment with 10, 20, and 40 Mg/d in Patients with Schizophrenia. J. Clin. Psychopharmacol. 29, 278–283. 10.1097/jcp.0b013e3181a289cb 19440083

[B8] Clayton A. H. Alkis A. R. Parikh N. B. Votta J. G. (2016). Sexual Dysfunction Due to Psychotropic Medications. Psychiatr. Clin. North America 39, 427–463. 10.1016/j.psc.2016.04.006 27514298

[B9] Cowen P. J. Anderson I. M. Gartside S. E. (1990). Endocrinological Responses to 5-HT. Ann. NY Acad. Sci. 600, 250–257. discussion 257-259. 10.1111/j.1749-6632.1990.tb16887.x 2252313

[B10] Cutler A. J. (2003). Sexual Dysfunction and Antipsychotic Treatment. Psychoneuroendocrinology 28 (Suppl. 1), 69–82. 10.1016/s0306-4530(02)00113-0 12504073

[B11] Daimon M. Kamba A. Murakami H. Mizushiri S. Osonoi S. Yamaichi M. (2017). Association between Serum Prolactin Levels and Insulin Resistance in Non-diabetic Men. PLoS One 12, e0175204. 10.1371/journal.pone.0175204 28384295 PMC5383244

[B12] De Boer M. K. Castelein S. Wiersma D. Schoevers R. A. Knegtering H. (2015). The Facts about Sexual (Dys)function in Schizophrenia: an Overview of Clinically Relevant Findings. Schizophrenia Bull. 41, 674–686. 10.1093/schbul/sbv001 PMC4393701 25721311

[B13] De Hert M. Detraux J. Peuskens J. (2014). Second-generation and Newly Approved Antipsychotics, Serum Prolactin Levels and Sexual Dysfunctions: a Critical Literature Review. Expert Opin. Drug Saf. 13, 605–624. 10.1517/14740338.2014.906579 24697217

[B14] Dehelean L. Romosan A. M. Papava I. Bredicean C. A. Dumitrascu V. Ursoniu S. (2020). Prolactin Response to Antipsychotics: An Inpatient Study. PLoS One 15, e0228648. 10.1371/journal.pone.0228648 32017792 PMC6999917

[B15] Di Francesco S. Caruso M. Robuffo I. Militello A. Toniato E. (2019). The Impact of Metabolic Syndrome and its Components on Female Sexual Dysfunction: A Narrative Mini-Review. Curr. Urol. 12, 57–63. 10.1159/000489420 31114461 PMC6504795

[B16] Dumontaud M. Korchia T. Khouani J. Lancon C. Auquier P. Boyer L. (2020). Sexual Dysfunctions in Schizophrenia: Beyond Antipsychotics. A Systematic Review. Prog. Neuro-Psychopharmacology Biol. Psychiatry 98, 109804. 10.1016/j.pnpbp.2019.109804 31711954

[B17] Fanta T. Haile K. Abebaw D. Assefa D. Hibdye G. (2018). Assessment of Sexual Dysfunction and Associated Factors Among Patients with Schizophrenia in Ethiopia, 2017. BMC Psychiatry 18, 158. 10.1186/s12888-018-1738-3 29843656 PMC5975573

[B18] Finn S. E. Bailey J. M. Schultz R. T. Faber R. (1990). Subjective Utility Ratings of Neuroleptics in Treating Schizophrenia. Psychol. Med. 20, 843–848. 10.1017/s0033291700036539 1980954

[B19] Fujii A. Yasui-Furukori N. Sugawara N. Sato Y. Nakagami T. Saito M. (2010). Sexual Dysfunction in Japanese Patients with Schizophrenia Treated with Antipsychotics. Prog. Neuro-Psychopharmacology Biol. Psychiatry 34, 288–293. 10.1016/j.pnpbp.2009.11.022 19951735

[B20] Gragnoli C. Reeves G. M. Reazer J. Postolache T. T. (2016). Dopamine-prolactin Pathway Potentially Contributes to the Schizophrenia and Type 2 Diabetes Comorbidity. Transl Psychiatry 6, e785. 10.1038/tp.2016.50 27093067 PMC4872408

[B21] Grigg J. Worsley R. Thew C. Gurvich C. Thomas N. Kulkarni J. (2017). Antipsychotic-induced Hyperprolactinemia: Synthesis of World-wide Guidelines and Integrated Recommendations for Assessment, Management and Future Research. Psychopharmacology 234, 3279–3297. 10.1007/s00213-017-4730-6 28889207

[B22] Häfner H. (2002). Schizophrenia: Do Men and Women Suffer from the Same Disease? Rev. Psiquiatr. Clín. 29, 267–292. 10.1590/s0101-60832002000600002

[B23] Hirsch L. Yang J. Bresee L. Jette N. Patten S. Pringsheim T. (2017). Second-Generation Antipsychotics and Metabolic Side Effects: A Systematic Review of Population-Based Studies. Drug Saf. 40, 771–781. 10.1007/s40264-017-0543-0 28585153

[B24] Huang Y.-H. Hou C.-L. Ng C. H. Chen X. Wang Q.-W. Huang Z.-H. (2019). Sexual Dysfunction in Chinese Rural Patients with Schizophrenia. BMC Psychiatry 19, 218. 10.1186/s12888-019-2205-5 31299942 PMC6624902

[B25] Huhn M. Nikolakopoulou A. Schneider-Thoma J. Krause M. Samara M. Peter N. (2019). Comparative Efficacy and Tolerability of 32 Oral Antipsychotics for the Acute Treatment of Adults with Multi-Episode Schizophrenia: a Systematic Review and Network Meta-Analysis. The Lancet 394, 939–951. 10.1016/s0140-6736(19)31135-3 PMC6891890 31303314

[B26] Johnsen E. Kroken R. Løberg E. M. Kjelby E. Jørgensen H. A. (2011). Sexual Dysfunction and Hyperprolactinemia in Male Psychotic Inpatients: a Cross-Sectional Study. Adv. Urol. 2011, 686924. 10.1155/2011/686924 22190916 PMC3235493

[B27] Karagianis J. L. Baksh A. (2003). High-dose Olanzapine and Prolactin Levels. J. Clin. Psychiatry 64, 1192–1194. 10.4088/jcp.v64n1008 14658967

[B28] Kay S. R. Fiszbein A. Opler L. A. (1987). The Positive and Negative Syndrome Scale (PANSS) for Schizophrenia. Schizophrenia Bull. 13, 261–276. 10.1093/schbul/13.2.261 3616518

[B29] Kheng Yee O. Muhd Ramli E. R. Che Ismail H. (2014). Remitted Male Schizophrenia Patients with Sexual Dysfunction. J. Sex. Med. 11, 956–965. 10.1111/jsm.12246 23845160

[B30] Kikuchi T. Iwamoto K. Sasada K. Aleksic B. Yoshida K. Ozaki N. (2012). Sexual Dysfunction and Hyperprolactinemia in Japanese Schizophrenic Patients Taking Antipsychotics. Prog. Neuro-Psychopharmacology Biol. Psychiatry 37, 26–32. 10.1016/j.pnpbp.2011.11.016 22172534

[B31] Kinon B. J. Gilmore J. A. Liu H. Halbreich U. M. (2003). Hyperprolactinemia in Response to Antipsychotic Drugs: Characterization across Comparative Clinical Trials. Psychoneuroendocrinology 28 (Suppl. 2), 69–82. 10.1016/s0306-4530(02)00128-2 12650682

[B32] Kinon B. J. Volavka J. Stauffer V. Edwards S. E. Liu-Seifert H. Chen L. (2008). Standard and Higher Dose of Olanzapine in Patients with Schizophrenia or Schizoaffective Disorder. J. Clin. Psychopharmacol. 28, 392–400. 10.1097/jcp.0b013e31817e63a5 18626265

[B33] Lee J.-Y. Kim S.-W. Lee Y.-H. Kang H.-J. Kim S.-Y. Bae K.-Y. (2015). Factors Associated with Self-Rated Sexual Function in Korean Patients with Schizophrenia Receiving Risperidone Monotherapy. Hum. Psychopharmacol. Clin. Exp. 30, 416–424. 10.1002/hup.2489 26123060

[B34] Lewis R. W. Fugl‐Meyer K. S. Bosch R. Fugl‐Meyer A. R. Laumann E. O. Lizza E. (2004). Epidemiology/risk Factors of Sexual Dysfunction. J. Sex. Med. 1, 35–39. 10.1111/j.1743-6109.2004.10106.x 16422981

[B35] Lu M.-L. Chen C.-H. Kuo P.-T. Lin C.-H. Wu T.-H. (2018). Application of Plasma Levels of Olanzapine and N -Desmethyl-Olanzapine to Monitor Metabolic Parameters in Patients with Schizophrenia. Schizophrenia Res. 193, 139–145. 10.1016/j.schres.2017.07.022 28720417

[B36] Macotela Y. Triebel J. Clapp C. (2020). Time for a New Perspective on Prolactin in Metabolism. Trends Endocrinol. Metab. 31, 276–286. 10.1016/j.tem.2020.01.004 32044206

[B37] Maiorino M. I. Bellastella G. Giugliano D. Esposito K. (2018). From Inflammation to Sexual Dysfunctions: a Journey through Diabetes, Obesity, and Metabolic Syndrome. J. Endocrinol. Invest. 41, 1249–1258. 10.1007/s40618-018-0872-6 29549630

[B38] Martín J. C. Acuña M. J. Labrador J. Blanco M. Casas C. (2018). Sexual Dysfunction Factors in Patients with Schizophrenia Treated with Second Generation Antipsychotics: Not Only Prolactin. Actas Esp Psiquiatr 46, 217–225. 30552811

[B39] Matthews D. R. Hosker J. P. Rudenski A. S. Naylor B. A. Treacher D. F. Turner R. C. (1985). Homeostasis Model Assessment: Insulin Resistance and ?-cell Function from Fasting Plasma Glucose and Insulin Concentrations in Man. Diabetologia 28, 412–419. 10.1007/bf00280883 3899825

[B40] Mcgahuey C. A. Gelenberg A. J. Laukes C. A. Moreno F. A. Delgado P. L. Mcknight K. M. (2000). The Arizona Sexual Experience Scale (ASEX): Reliability and Validity. J. Sex. Marital Ther. 26, 25–40. 10.1080/009262300278623 10693114

[B41] Muniyappa R. Sowers J. R. (2013). Role of Insulin Resistance in Endothelial Dysfunction. Rev. Endocr. Metab. Disord. 14, 5–12. 10.1007/s11154-012-9229-1 23306778 PMC3594115

[B42] Nicolosi A. Glasser D. B. Kim S. C. Marumo K. Laumann E. O. Group G. I. (2005). Sexual Behaviour and Dysfunction and Help-Seeking Patterns in Adults Aged 40-80 Years in the Urban Population of Asian Countries. BJU Int. 95, 609–614. 10.1111/j.1464-410x.2005.05348.x 15705089

[B43] Petruzzelli M. G. Margari M. Peschechera A. De Giambattista C. De Giacomo A. Matera E. (2018). Hyperprolactinemia and Insulin Resistance in Drug Naive Patients with Early Onset First Episode Psychosis. BMC Psychiatry 18, 246. 10.1186/s12888-018-1827-3 30068291 PMC6090964

[B44] Peuskens J. Pani L. Detraux J. De Hert M. (2014). The Effects of Novel and Newly Approved Antipsychotics on Serum Prolactin Levels: a Comprehensive Review. CNS Drugs 28, 421–453. 10.1007/s40263-014-0157-3 24677189 PMC4022988

[B45] Rahmanian E. Salari N. Mohammadi M. Jalali R. (2019). Evaluation of Sexual Dysfunction and Female Sexual Dysfunction Indicators in Women with Type 2 Diabetes: a Systematic Review and Meta-Analysis. Diabetology Metab. Syndr. 11, 73. 10.1186/s13098-019-0469-z PMC6712652 31467595

[B46] Rubio-Abadal E. Del Cacho N. Saenz-Navarrete G. Arranz B. Cambra R.-M. Cuadras D. (2016). How Hyperprolactinemia Affects Sexual Function in Patients under Antipsychotic Treatment. J. Clin. Psychopharmacol. 36, 422–428. 10.1097/jcp.0000000000000539 27433851

[B47] Schulster M. L. Liang S. E. Najari B. B. (2017). Metabolic Syndrome and Sexual Dysfunction. Curr. Opin. Urol. 27, 435–440. 10.1097/mou.0000000000000426 28650864

[B48] Serretti A. Chiesa A. (2011). A Meta-Analysis of Sexual Dysfunction in Psychiatric Patients Taking Antipsychotics. Int. Clin. Psychopharmacol. 26, 130–140. 10.1097/yic.0b013e328341e434 21191308

[B49] Simiyon M. Chandra P. S. Desai G. (2016). Sexual Dysfunction Among Women with Schizophrenia-A Cross Sectional Study from India. Asian J. Psychiatry 24, 93–98. 10.1016/j.ajp.2016.08.022 27931918

[B50] Smith S. Wheeler M. J. Murray R. O’Keane V. (2002). The Effects of Antipsychotic-Induced Hyperprolactinaemia on the Hypothalamic-Pituitary-Gonadal Axis. J. Clin. Psychopharmacol. 22, 109–114. 10.1097/00004714-200204000-00002 11910254

[B51] Souaiby L. Kazour F. Zoghbi M. Bou Khalil R. Richa S. (2019). Sexual Dysfunction in Patients with Schizophrenia and Schizoaffective Disorder and its Association with Adherence to Antipsychotic Medication. J. Ment. Health, 29 1–8. 10.1080/09638237.2019.1581333 30862199

[B52] Suzuki Y. Ono S. Sugai T. Fukui N. Watanabe J. Tsuneyama N. (2011). Dose-dependent Effects of Olanzapine on QT Intervals and Plasma Prolactin Levels in Japanese Patients with Stable Schizophrenia. Hum. Psychopharmacol. 26, 440–443. 10.1002/hup.1218 21823168

[B53] Takeuchi H. Suzuki T. Remington G. Watanabe K. Mimura M. Uchida H. (2014). Lack of Effect of Risperidone or Olanzapine Dose Reduction on Metabolic Parameters, Prolactin, and Corrected QT Interval in Stable Patients with Schizophrenia. J. Clin. Psychopharmacol. 34, 517–520. 10.1097/jcp.0000000000000142 24911439

[B54] Tan C.-E. Ma S. Wai D. Chew S.-K. Tai E.-S. (2004). Can We Apply the National Cholesterol Education Program Adult Treatment Panel Definition of the Metabolic Syndrome to Asians?. Diabetes Care 27, 1182–1186. 10.2337/diacare.27.5.1182 15111542

[B55] Wallace I. R. Mckinley M. C. Bell P. M. Hunter S. J. (2013). Sex Hormone Binding Globulin and Insulin Resistance. Clin. Endocrinol. 78, 321–329. 10.1111/cen.12086 23121642

[B56] Yasui-Furukori N. Saito M. Nakagami T. Sugawara N. Sato Y. Tsuchimine S. (2010). Gender-specific Prolactin Response to Antipsychotic Treatments with Risperidone and Olanzapine and its Relationship to Drug Concentrations in Patients with Acutely Exacerbated Schizophrenia. Prog. Neuro-Psychopharmacology Biol. Psychiatry 34, 537–540. 10.1016/j.pnpbp.2010.02.014 20170699

[B57] Zhao S. Wang X. Qiang X. Wang H. He J. Shen M. (2020). Is There an Association between Schizophrenia and Sexual Dysfunction in Both Sexes? A Systematic Review and Meta-Analysis. J. Sex. Med. 17, 1476–1488. 10.1016/j.jsxm.2020.03.005 32299716

