# Supplementary material for: The Relationships Between Hyperprolactinemia, Metabolic Disturbance, and Sexual Dysfunction in Patients With Schizophrenia Under Olanzapine Treatment
Source: Front Pharmacol. 2021 Aug 5;12:718800. doi: 10.3389/fphar.2021.718800 (PMC8374865; doi:10.3389/fphar.2021.718800)
Supplement: Supplementary file 1 [file Table1.DOCX]

**Supplementary Table 1. Demographic, clinical and laboratory characteristics of study subjects by sex.**

|  | Male (n=132) | Female (n=147) | P value |
| --- | --- | --- | --- |
| **Demographic parameters** |  |  |  |
| Age (years) | 41.6 ± 11.8 | 43.9 ± 11.2 | 0.101 |
| Duration of illness (years) | 14.1 ± 9.0 | 16.0 ± 10.2 | 0.090 |
| Tobacco use | 51.5% | 13.6% | <0.001 |
| Marital status |  |  |  |
| Married/With partner | 19 | 42 | 0.006 |
| Single/Divorced/Widowed | 113 | 105 |  |
| BMI | 25.8 ± 5.2 | 25.6 ± 4.4 | 0.714 |
| Waist circumference (cm) | 90.6 ± 12.1 | 85.5 ± 11.2 | <0.001 |
| SBP (mmHg) | 123.6 ± 14.3 | 118.3 ± 14.5 | 0.002 |
| DBP (mmHg) | 77.9 ± 10.0 | 75.0 ± 10.9 | 0.024 |
| **Concomitant medication** |  |  |  |
| Benzodiazepine | 45.5% | 46.9% | 0.811 |
| Anticholinergics | 25.0% | 31.3% | 0.287 |
| Antidepressant | 8.3% | 11.6% | 0.428 |
| **Clinical parameters** |  |  |  |
| Olanzapine dose | 14.2 ± 5.8 | 14.1 ± 5.2 | 0.792 |
| PANSS positive score | 15.5 ± 5.5 | 15.3 ± 5.3 | 0.723 |
| PANSS negative score | 15.7 ± 5.3 | 15.6 ± 5.6 | 0.909 |
| PANSS general score | 27.2 ± 7.4 | 27.1 ± 8.1 | 0.912 |
| PANSS total score | 58.4 ± 14.5 | 58.0 ± 16.1 | 0.825 |
| ASEX score | 16.6 ± 5.3 | 19.8 ± 6.5 | <0.001 |
| Sexual dysfunction | 40.9% | 65.3% | <0.001 |
| **Laboratory parameters** |  |  |  |
| Prolactin level (ng/mL) | 21.0 ± 21.7 | 29.2 ± 22.5 | 0.002 |
| Hyperprolactinemia | 50.8% | 52.4% | 0.811 |
| Glucose level (mg/dL) | 90.1 ± 22.1 | 99.0 ± 36.0 | 0.013 |
| Insulin level (μU/mL) | 13.9 ± 17.5 | 11.9 ± 9.9 | 0.252 |
| HOMA-IR | 3.6 ± 6.3 | 2.9 ± 2.6 | 0.231 |
| Triglycerides (mg/dL) | 165.5 ± 98.2 | 146.6 ± 97.2 | 0.108 |
| HDL-C (mg/dL) | 47.2 ± 17.4 | 52.1 ± 16.1 | 0.016 |
| Metabolic syndrome | 41.7% | 45.6% | 0.547 |

ASEX, Arizona Sexual Experiences Scale; BMI, body mass index; DBP, diastolic blood pressure; HDL-C, high-density lipoprotein cholesterol; HOMA-IR, homeostasis model assessment for insulin resistance; PANSS, Positive and Negative Syndrome Scale; SBP, systolic blood pressure.
